# Genomic data support the taxonomic validity of Middle American livebearers *Poeciliopsis gracilis* and *Poeciliopsis pleurospilus* (Cyprinodontiformes: Poeciliidae)

**DOI:** 10.1371/journal.pone.0262687

**Published:** 2022-01-31

**Authors:** Liam M. Ward, Caleb D. McMahan, Basanta Khakurel, April M. Wright, Kyle R. Piller

**Affiliations:** 1 Department of Biological Sciences, Southeastern Louisiana University, Hammond, Louisiana, United States of America; 2 Department of Biology, University of Oklahoma, Norman, Oklahoma, United States of America; 3 Field Museum of Natural History, Chicago, Illinois, United States of America; DePaul University, UNITED STATES

## Abstract

*Poeciliopsis* (Cyprinodontiformes: Poeciliidae) is a genus comprised of 25 species of freshwater fishes. Several well-known taxonomic uncertainties exist within the genus, especially in relation to the taxonomic status of *Poeciliopsis pleurospilus* and *P*. *gracilis*. However, to date, no studies have been conducted to specifically address the taxonomic status of these two species. The goal of this study was to examine the taxonomic validity of *P*. *pleurospilus* and *P*. *gracilis* using genomic data (ddRADseq) in phylogenetic, population genetic, and species delimitation frameworks. Multiple analyses support the recognition of both taxa as distinct species and also permits us to revise their respective distributions. A species delimitation analysis indicates that *P*. *pleurospilus* and *P*. *gracilis* are distinct species, each of which consists of two distinct lineages that are geographically structured. Phylogenetic and population genetic analyses provide clear evidence that individuals of *P*. *gracilis* are distributed north and west of the Isthmus of Tehuantepec in both Pacific and Atlantic river systems in Mexico, whereas individuals of *P*. *pleurospilus* are distributed in both Atlantic and Pacific river systems south and east of the Isthmus of Tehuantepec, from southern Mexico to Honduras.

## Introduction

Middle America is a biologically and geologically diverse region that includes Mexico and the seven countries of Central America as well as the islands of the Greater Antilles [1, 2]. Various volcanic and tectonic events have shaped this dynamic landscape and have resulted in the formation of many geographic barriers, which have, in turn, affected the biodiversity of the region [3, 4]. For example, the closure of the narrow land-bridge at the Isthmus of Panama during the Pliocene had direct consequences for global ocean currents, thereby affecting global atmospheric circulation patterns and altering the tropical ecosystems of the region [5–9]. Another example is the formation of the Trans-Mexican Volcanic Belt (TMVB) beginning in the early Miocene and continuing into the present day, with large changes in topography occurring over the past 3 Myr, including the formation of some presently-active volcanos [10]. The TMVB has been demonstrated to be a substantial barrier to gene flow in populations of freshwater fishes in Mexico [11–13]. Additionally, various events such as stream capture, artificial modifications to stream flow, and sea-level rise have disconnected streams and directly limited or enhanced the dispersal ability of freshwater fishes [14–22]. These issues have been highlighted by studies of freshwater fish diversity, which have played an important role in understanding the biogeographic history of Middle America [21–29].

One of the most ubiquitous groups of freshwater fishes in Middle America is the Poeciliidae [30], a family of New World cyprinodontiform fishes [31]. Poeciliids have been broadly studied in vicariance biogeography due to parallel patterns of distributions being noted for some fishes, reptiles, and plants [16]. In addition, much is known about the phylogenetic relationships within Poeciliidae [21, 32–38]. The family currently consists of 276 species in 27 recognized genera [34, 39, 40]. One of the more diverse genera in the family, *Poeciliopsis* [41], consists of 25 species [36, 42]. The genus inhabits a wide range of environments from lowlands to highlands, in rivers and streams, lakes, and springs, and it is primarily distributed in Pacific-slope drainages of Middle America [26, 43–45], but several species have been documented in Atlantic basin streams [44–46]. Although the genus has been heavily studied from biogeographic and phylogenetic perspectives [40, 42, 46–50], many species have been excluded from phylogenetic analyses. These exclusions are due to several well-known taxonomic uncertainties within *Poeciliopsis* [45], especially in relation to the status of *Poeciliopsis gracilis* [51] and *P*. *pleurospilus* [52].

**Fig 1:**
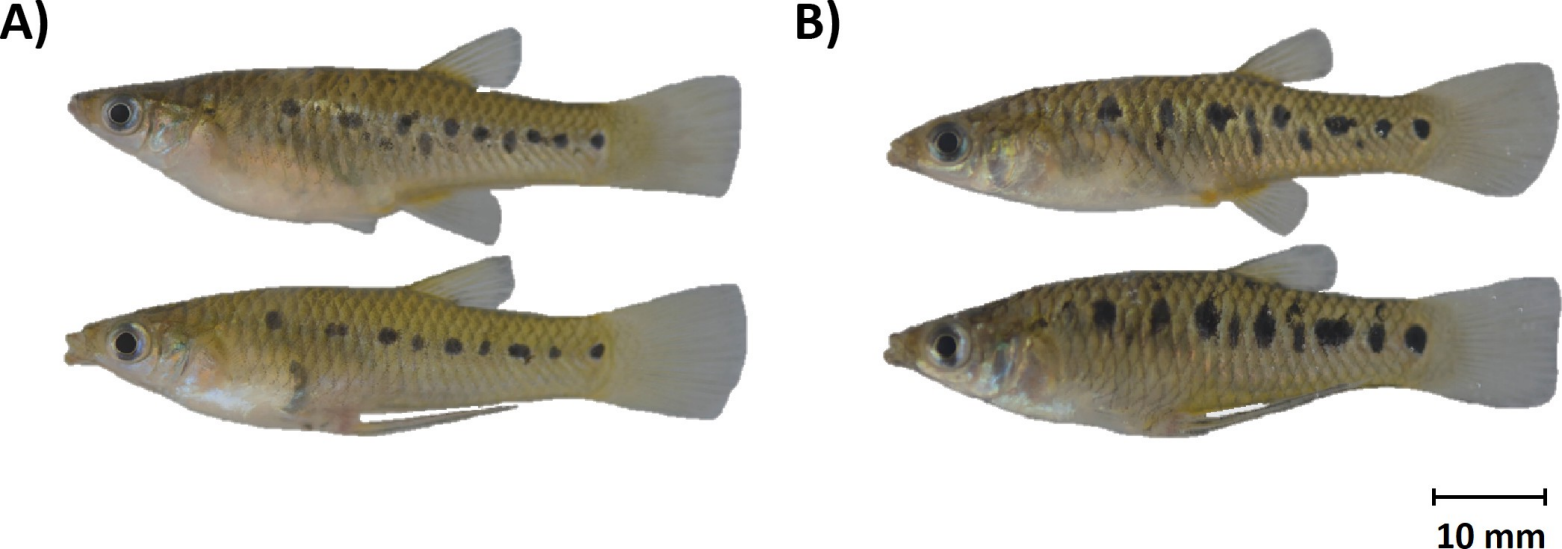
Images of *Poecilopsis gracilis* and *Poeciliopsis pleurospilus*. **A)** Female (upper) and male (lower) specimens of *P*. *gracilis* from the Río Ajal in Oaxaca, Mexico. **B)** Female (upper) and male (lower) specimens of *P*. *pleurospilus* from the Río Ostuta in Oaxaca, Mexico.

*Poeciliopsis gracilis* was originally described as *Xiphophorus gracilis* from the Río Orizaba on the Atlantic Slope of Veracruz, Mexico [51]. Historically, *P*. *gracilis* has been treated as a synonym of *P*. *pleurospilus* [43, 53, 54]; however, Miller et al. [45] identified a distinction between the species based on the separation of their native distributions as well as variation in pigmentation. Individuals of *P*. *gracilis* are recognized by lateral markings that are small spots no larger than the diameter of the pupil, or more frequently, horizontal dashes that are sometimes doubled or fused (Fig 1A) [45]. Native populations of *P*. *gracilis* are hypothesized to be restricted to the Atlantic-slope rivers in the states of Veracruz and Oaxaca, Mexico, as well as having been introduced in the Ríos Pánuco (Atlantic) and Balsas (Pacific) basins within Mexico [45].

*Poeciliopsis pleurospilus* was originally described as *Girardinus pleurospilus* from Lago Dueñas in Guatemala (Pacific Basin). Individuals of *P*. *pleurospilus* are recognized by lateral markings that are large, dark, oval spots, which are larger than the diameter of the pupil, in addition to sometimes possessing one to three crescent-shaped bars along the lateral flank of the body (Fig 1B) [45]. In the current literature, native populations of *P*. *pleurospilus* are hypothesized to inhabit Pacific-slope systems from Mexico to Honduras, as well as Atlantic-slope systems including the Río Grijalva (Mexico and Guatemala), upper Río Motagua (Guatemala), and Río Ulua (Honduras) [26, 45, 55].

Due to the fact that *P*. *gracilis* and *P*. *pleurospilus* are morphologically and ecologically similar species, most phylogenetic studies have only included one or the other, but not both [11, 42, 46, 56]. To date, no studies have been conducted to specifically address the taxonomic and phylogenetic status of these two species. The goal of this study was to examine the taxonomic validity of *P*. *gracilis* and *P*. *pleurospilus* using genomic-scale DNA sequence data. We tested the monophyly of each species by collecting double digest restriction-site associated DNA sequencing data (ddRADseq) [57] from multiple populations and localities across their putative ranges. The ddRADseq data provided a robust multilocus data set from which we were able to perform Maximum Likelihood (ML) phylogenetic inference, which allowed us to infer the evolutionary relationships among populations of each species sampled across their respective ranges in Mexico, Guatemala, El Salvador, and Honduras [26, 45, 55, 58]. We also performed several population genetic analyses to examine the amount of genetic diversity and genetic structure within and among the recovered lineages. In addition, we conducted a species delimitation analysis to inform our conclusions on diversity among the various populations. The results from this study provide much-needed insight into the long-standing taxonomic issue of the taxonomic distinctiveness of these two morphologically similar congeners.

## Materials and methods

### Taxon sampling

One-hundred forty-eight individuals were used in this study after filtering samples for low call quality, including 134 individuals currently recognized as either *P*. *gracilis* or *P*. *pleurospilus* (S1 Table), following the characters presented in Miller et. al. [45]. Thirteen samples from three other species of *Poeciliopsis* (*P*. *fasciata*, n = 5*; P*. *infans*, n = 4; *P*. *turrubarensis*, n = 4), and a single outgroup *Brachyrhaphis rhabdophora*, also were included in the study (S2 Table). Tissue samples (fin clips) were collected from various localities in Oaxaca and Veracruz, Mexico in November, 2019 using a standard 10’ x 6’ seine, and immediately preserved in 95% ethanol (approved by Southeastern Louisiana University IACUC Committee Protocol #0074). Additional specimens from river systems in Mexico, Guatemala, El Salvador, and Honduras were obtained from the tissue collections at Southeastern Louisiana University (SLU), the Field Museum of Natural History (FMNH), Louisiana State University Museum of Natural Science (LSUMZ), and the Universidad de Ciencias y Artes de Chiapas Museo de Zoología (UNICACH). Specific locality information for each specimen currently recognized as *P*. *gracilis* and *P*. *pleurospilus* is included in S1 Table.

### Laboratory methods

DNA was extracted from fin clips using the Qiagen DNeasy Tissue Extraction Kit following the manufacturer’s recommendations. Three double digest restriction enzyme DNA (ddRAD) libraries (batch information available in S3 Table) were prepared following a modified version (S1 Appendix) of the protocol from Peterson et al. [57]. Each sample was digested with MspI and PstI restriction enzymes and ligated to common (5’- GTGACTGGAGTTCAGACGTGTGCTCTTCCGATCT—3’) and unique oligos (S3 Table).

We then used a BluePippin machine to size select for 300–500 bp fragments, then libraries were sent to the University of Oregon’s Genomic and Cell Characterization Core Facility (GC3F) for Illumina Sequencing on the Hiseq 4000 for 100 bp single-end reads.

### Bioinformatics

The raw data files returned from GC3F were run through FastQC v0.11.3 [59] to check the overall quality of the reads from the Illumina run. The FastQ file output from the previous step was input into the ipyrad [60] pipeline for assembly and initial filtering (parameters in S4 Table). Reads that contained more than 5 bases with a low-quality Phred score (<33) were excluded. Reads were then clustered based on an 85% similarity threshold and reads with less than 6x coverage were filtered out. A maximum of 5 ambiguous base calls and 5 heterozygous sites per read were allowed during filtering. Summary statistics of the ipyrad assembly are available in S2 Appendix.

Additional filtering using VCFtools [61] excluded individuals with more than 95% missing data, and single nucleotide polymorphism (SNP) loci with a 60% call rate or lower. We retained one dataset that consisted of all specimens that was used for our phylogenetic inference. A separate dataset that consisted only of ingroup individuals was used for the population genetic analyses. Aligned datasets, tree file, laboratory protocols, and bioinformatic workflow available in a Data Dryad accession (doi:10.5061/dryad.mkkwh711m).

### Phylogenetic inference

Concatenated ddRAD loci were analyzed under Maximum Likelihood using IQ-TREEv1.6.12 [62]. The filtered VCF file was converted to FASTA format using PGDSpider v2.1.1.5 [63] and input into Model Finder [57, 64] within IQ-TREE to compute the log-likelihood of an initial parsimony tree for many different models under the Akaike information criterion (AIC), corrected Akaike information criterion (AICc), and the Bayesian information criterion (BIC). The AIC, AICc, and BIC output all selected GTR+F+R2 as the best fit model for our data. Ultrafast bootstraps were run for 10,000 generations and a Shimodaira-Hasegawa-like approximate likelihood ratio test (SH-aLRT) was run for 1,000 replicates in IQ-TREE and the resulting tree was visualized using FigTree v1.4.4 [65].

### Population structure

We used VCFtools to exclude individuals with more than 95% missing data, loci (SNPs) with a 60% call rate or lower, along with excluding all outgroup individuals to produce a VCF file of raw reads for 134 samples of *P*. *pleurospilus* and *P*. *gracilis*. We then used RStudio 4.0.0 [66] to attach relevant population information and conduct population genetic analyses using the R package adegenet v 2.0.0 [67]. The first analysis was a Discriminant Analysis of Principal Components (DAPC) [68] following the methods of Grünwald et al. [69] and the vignette provided by Jombart and Collins 2015 [67]. We defined groups *a priori* and determined the optimal number of Principal Components (PCs) to retain using the a-score metric (S1 Fig) built into adegenet [67], which led us to include six PCs and three discriminant functions. We used the posterior assignment of each sample assigned within the DAPC object to visualize a composite stacked bar plot following the methods of Grünwald et al. [70]. Finally, we calculated an unweighted Weir and Cockerham [71] pairwise F_ST_ between clades of ingroup individuals using VCFtools. Bioinformatic pipeline for all phylogenetic and population genetic analyses available in S3 Appendix.

### Species delimitation analyses

In order to assign a marginal probability to our hypotheses of species delimitation, we used the software BPP [72, 73]. We performed this analysis on the concatenated SNP file obtained in the *Bioinformatics* section above, with one outgroup sequence, *Brachyrhaphis rhabdophora*. BPP uses the multi-species coalescent to estimate divergence times and population sizes for both extant and ancestral sequences. This information is used to derive the probability that different groups of organisms are actually separate populations. We used the algorithm referred to as “A10”, in which the researcher provides a guide species tree to be tested. We tested three population structure hypotheses including: 1) the one-species model, in which all in-group taxa are one species, 2) the two-species model in which the PM + AM populations are one species (*P*. *gracilis*) and the PSM + MCA populations are a separate species (*P*. *pleurospilus*) and 3) the four-species model, in which PM, AM, PSM, MCA are all separate species.

In order to estimate a marginal probability for the data under each model (i.e., one not biased by MCMC hill-climbing), we used the stepping stone method [74]. We did this following the stepping stone procedure described in Rannala and Yang [72] as implemented in the bppr R package [75]. Each stepping stone for each of the three models was run to convergence, as assessed in the bppr package, and Bayes Factor model comparisons were performed in bppr.

## Results

### Phylogenetic inference

**Fig 2:**
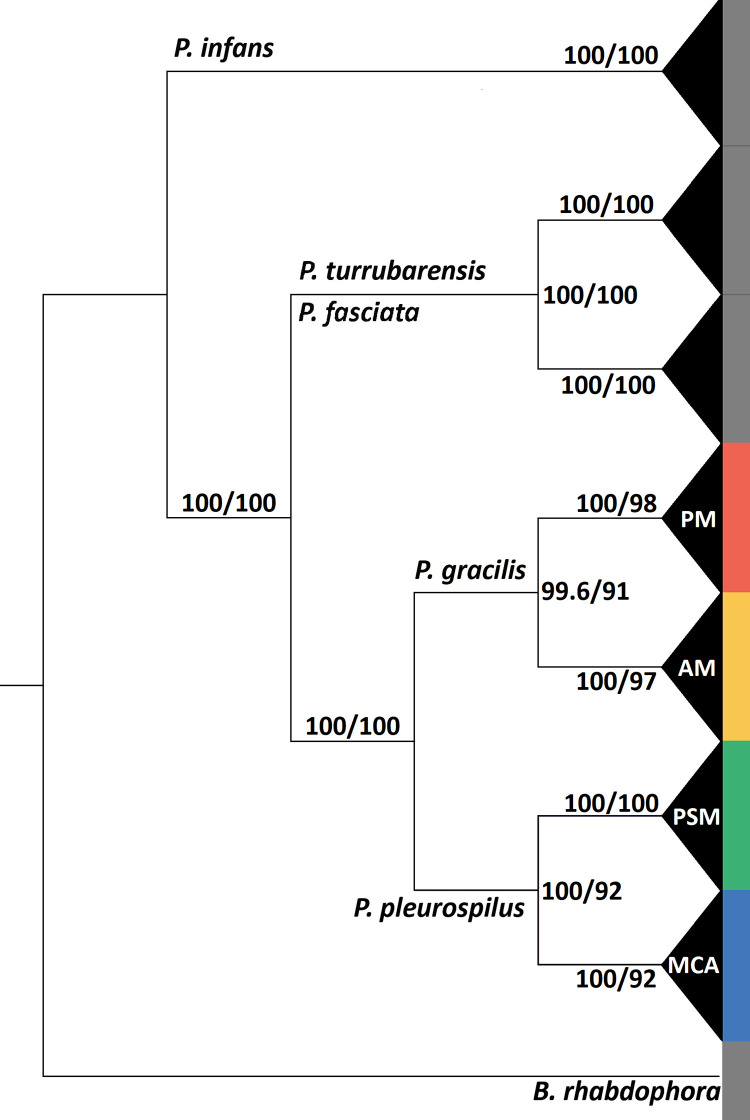
Collapsed ddRADseq cladogram with SH-aLRT (left) and ultrafast bootstrap (right) values. Colorized clades indicate *P*. *gracilis* (PM; AM) and *P*. *pleurospilus* (PSM; MCA).

**Fig 3:**
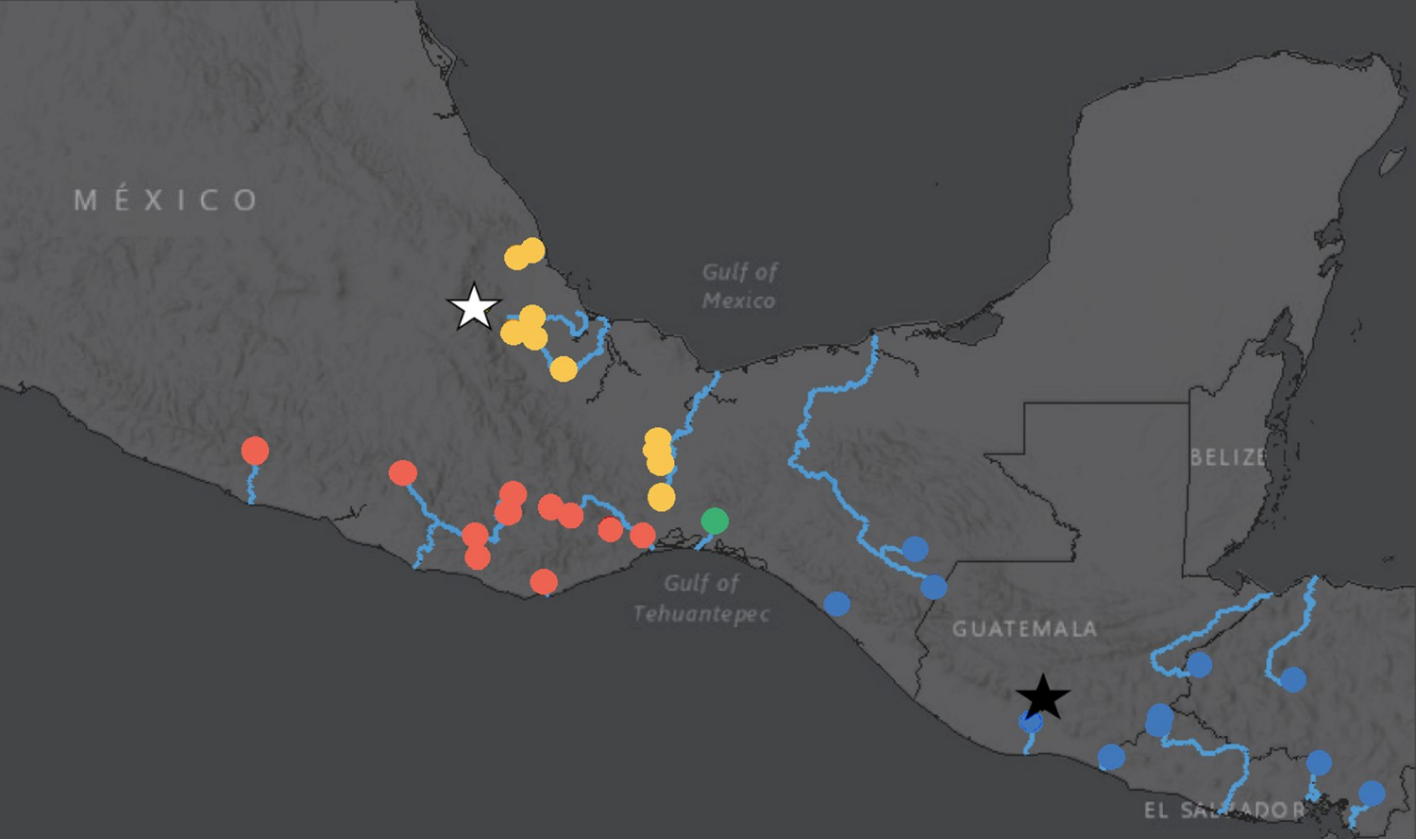
Distribution map of sampled localities. Red circles represent Pacific Mexico (PM); orange circles represent Atlantic Mexico (AM); green circles represent Pacific Southern Mexico (PSM); and blue circles represent Mexico and Central America (MCA). The white star; represents the type locality of *P*. *gracilis*; the black star represents the type locality of *P*. *pleurospilus*. Map made in ArcMap 10.8.1 using the World Dark Gray Base map [76].

The full dataset including outgroups retained 178,130 out of a possible 1,817,209 SNPs. Additional read information is available in S4 Table. This dataset was used to infer a Maximum Likelihood phylogenetic tree under the GTR+F+R2 model. A collapsed cladogram rooted with *Brachyrhaphis rhabdophora* inferred seven major clades, four of which represented ingroup taxa (Fig 2). We found that the populations represented in the four major ingroup clades of our Maximum Likelihood inference (Fig 2) clustered into cohesive, allopatric groups that were geographically separate from other such groups. Specifically, we identified a ‘Pacific Mexico’ (PM) clade consisting of samples from populations from the Pacific-draining basins of southwestern Mexico; an ‘Atlantic Mexico’ (AM) clade from the Atlantic-draining basins of eastern Mexico; a ‘Pacific Southern Mexico’ (PSM) clade comprised of individuals collected from the Río Ostuta of southern Mexico; and an ‘Mexico and Central America’ (MCA) clade of individuals from Mexican drainage systems south of the Isthmus of Tehuantepec (including the Río Grijalva and nearby basins) and populations extending southward into El Salvador, Guatemala, and Honduras in Central America (Fig 3). Additional information on sampling localities is provided in S1 Table. Tree file with SH-aLRT and ultrafast bootstrap values available in supplementary material.

Four individuals of *Poeciliopsis infans* sampled from Manantial Mintzita, Michoacán, Mexico were sister to all other samples of *Poeciliopsis* included in this study, with 100% ultrafast bootstrap and 100% SH-aLRT support values (Fig 2). Four individuals of *P*. *turrubarensis* collected from the Ríos de la Virgen, Tehuantepec, Tequesistlan, and Totolapan, from the Pacific coast of Oaxaca, Mexico and five individuals of *P*. *fasciata* collected from the Río Ajal in Oaxaca, Mexico were sister to one another with 100% SH-aLRT and 100% ultrafast bootstrap support and sister to *P*. *gracilis* and *P*. *pleurospilus* (Fig 2).

**Fig 4:**
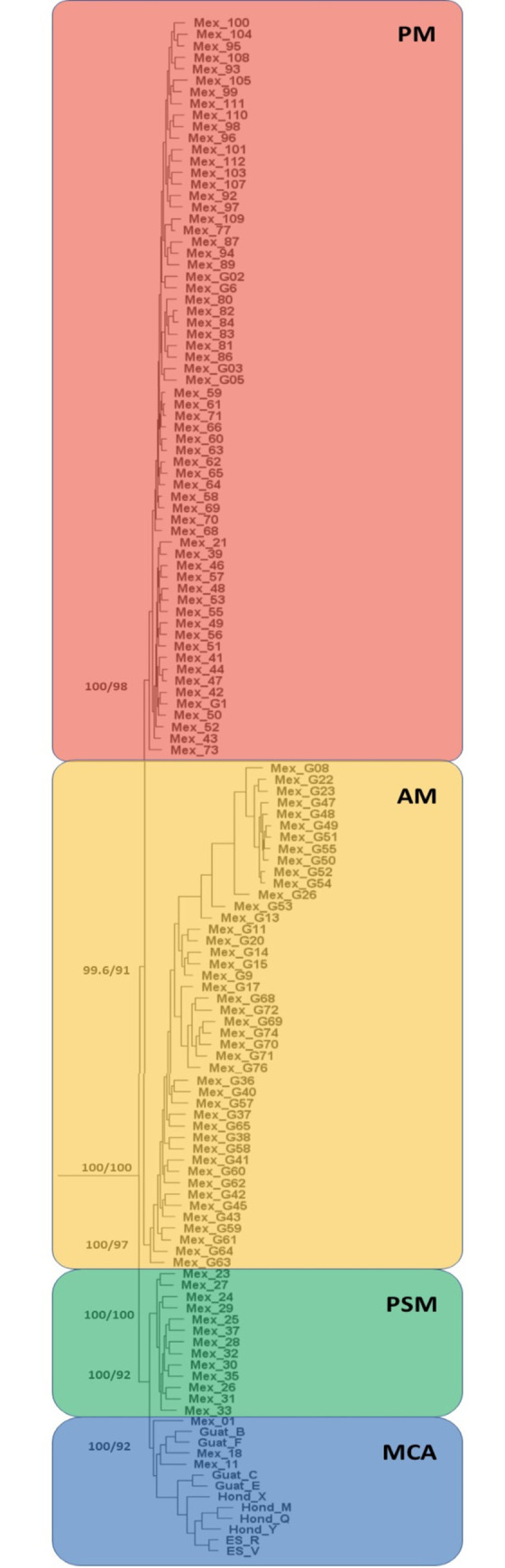
Phylogram depicting relationships among clades of *Poeciliopsis* ingroup individuals. Red indicates individuals from Pacific basin streams north and west of the Isthmus of Tehuantepec (PM); orange indicates individuals from Atlantic basin streams north and east of the Isthmus of Tehuantepec (AM); green indicates individuals from a Pacific basin stream (Río Ostuta) in Oaxaca, Mexico (PSM); and blue indicates individuals from both Pacific and Atlantic streams south of the Isthmus of Tehuantepec in Mexico, Guatemala, El Salvador, and Honduras (MCA). Branch support is indicated by SH-aLRT (left) and ultrafast bootstrap (right) values. Individuals identified in the field as *P*. *gracilis* based on pigmentation patterns are indicated by a “G” in the tip label.

Within the four ingroup clades, branch lengths were short and little geographic structure was recovered within any of the four clades, although clear geographic structure was seen among clades (Fig 4). The ultrafast bootstrap values are lower at terminal nodes within the ingroup clades compared to deeper nodes. Deeper splits among the four clades are supported by higher SH-aLRT (99.6–100) and higher ultrafast bootstrap values (91–100 UFBoot), signifying more robust clade assignments.

The PM clade consisted exclusively of specimens collected from Pacific-slope river systems (n = 64) (Fig 4). Individuals identified in the field as *P*. *pleurospilus* were collected from six localities in Oaxaca, Mexico, including the Ríos Coapa, de la Virgen, Octlan, Tehuantepec, Tequesistlan, and Totolapan. Also included in this clade were individuals identified in the field as *P*. *gracilis* that were collected from four localities in Oaxaca, Mexico, including the Ríos Huatulco, Las Flores, and Puente Tierra Azul, and Flor de Café; as well as an unnamed stream in Guerrero, Mexico. Branch lengths were extremely short within this clade, with ultrafast bootstrap support values ranging from 19%– 100% and SH-aLRT values ranging from 3.4–100%, indicating varying degrees of phylogenetic resolution in the data.

The AM clade consisted exclusively of specimens collected from Atlantic basin river systems (n = 44) (Fig 4) and were identified in the field as *P*. *gracilis*. Specimens were obtained from seven localities in Oaxaca, Mexico including three specimens from an unnamed arroyo, and specimens from the Ríos Ajal, Amapa, Barranca, Papaloapan, and two localities on the Río Tolosita; as well as from three localities in Veracruz, Mexico including the Ríos Actopan, Blanco, and Jaltepec. The type locality of *P*. *gracilis* is the Río Orizaba in Veracruz, Mexico (indicated by a white star in Fig 3), which is a tributary to the Río Blanco, indicating that this clade most likely represents individuals of topotypic *P*. *gracilis*. Ultrafast bootstrap support values ranged from 43% - 100% and SH-aLRT values ranged from 17.9–100%, indicating varying degrees of phylogenetic resolution within the clade.

The PSM clade consisted exclusively of specimens collected from the Río Ostuta (n = 13) in Oaxaca, Mexico (Pacific basin) (Fig 4). All individuals were identified in the field as *P*. *pleurospilus*. Branch lengths were short in this clade, and the bootstrap support values ranged from 33% - 100% and SH-aLRT values ranged from 27.5–100%, indicating varying degrees of phylogenetic resolution.

The MCA clade consisted of specimens collected from both the Pacific (n = 9) and Atlantic (n = 4) basin river systems (Fig 4). The Pacific basin specimens included individuals from Lago Güija and Laguna Metapán in El Salvador; individuals from the Río Achiguate and Río Negro in Guatemala; individuals from the Ríos Choluteca and Nacaome in Honduras; and the Río Margaritas in Chiapas, Mexico. The Atlantic basin specimens included individuals from the Ríos Amarillo and Tepemechin in Honduras; and the Ríos Ojo de Agua and Paso Hondo, in Chiapas, Mexico. The type locality of *P*. *pleurospilus* is Lago Dueñas on the Pacific slope of Guatemala. This lake no longer exists; however, the historical location of the lake is indicated by a black star (Fig 3). The historical location of Lago Dueñas is just under 12 km from the headwaters of the Río Achiguate, indicating that this clade most likely represents individuals of topotypic *P*. *pleurospilus*. Ultrafast bootstrap support values ranged from 87% - 100% and SH-aLRT values ranged from 70.2–100%, indicating relatively robust phylogenetic resolution within the clade.

### Population structure

**Fig 5:**
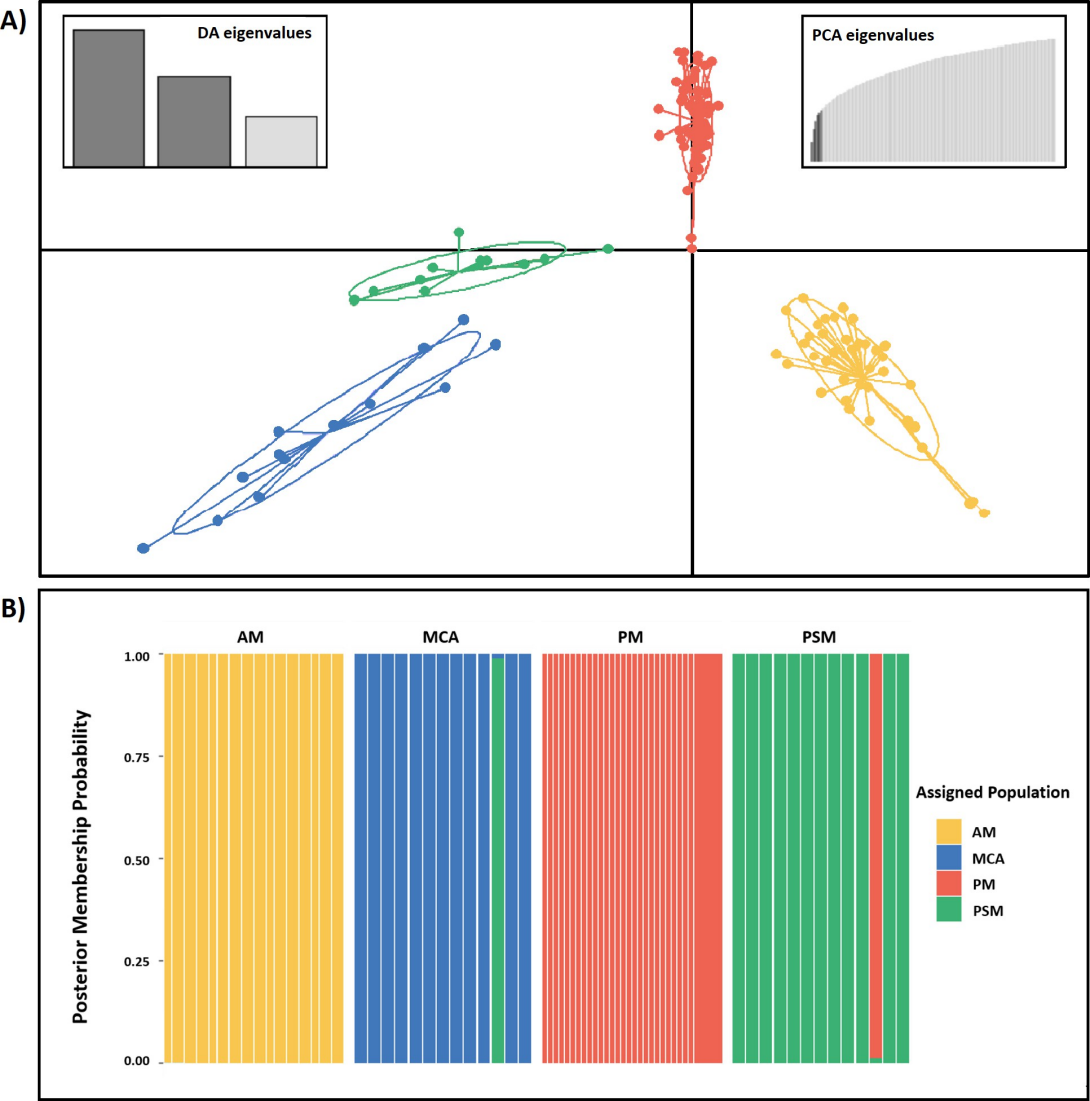
Discriminant Analysis of Principle Components (DAPC) and composite stacked bar plot. **A)** Discriminant Analysis of Principal Components (DAPC) of populations. Ellipses represent 95% confidence intervals. **B)** Composite Stacked Bar Plot of all individuals from the ingroup dataset. In panels A and B, inferred genetic clusters of individuals were correlated with our phylogenetic results (Figs 2 and 4) and assigned colors of the corresponding clade, as follows: PM cluster, red; AM cluster, orange; PSM cluster, green; MCA cluster, blue.

Various population genetic metrics were used to assess genetic variability among individuals and clusters of *P*. *gracilis* and *P*. *pleurospilus*. For these analyses we used a second dataset that excluded outgroup specimens. This dataset retained 213,593 out of a possible 1,817,209 sites. The first method employed was a DAPC, which recovered four distinct clusters. The 95% confidence ellipses showed no overlap among DAPC clusters, with the clustering showing varying distances from one another in the DAPC space (Fig 5A). The MCA and PSM clusters corresponded to samples collected south and east of the Isthmus of Tehuantepec and were the same individuals obtained in the MCA and PSM clades of our Maximum Likelihood phylogeny. The AM and PM clusters corresponded to samples collected from the Isthmus of Tehuantepec and north and east, and clustered more closely with one another than with either of the other populations.

We then used the DAPC object to visualize the population assignment of each sample and created a composite stacked bar plot (Fig 5B). In a stacked bar plot, the probability of population membership is illustrated on the y-axis, from 0 to 100% probability of belonging to a specific population. The x-axis contains a bin of each sample. Colors represent the pre-assigned populations by clade, whereas the groupings represent the populations inferred from the DAPC object. The composite stacked bar plots also recover the four distinct clusters revealed by the DAPC analysis. The PM and AM clusters are completely pure, with no admixture with any of the other clusters. The PSM cluster showed one individual admixed with the PM cluster and the MCA cluster showed one individual admixed with the PSM cluster.

An unweighted Weir & Cockerham pairwise F_ST_ was calculated between clusters of ingroup individuals, where values closer to zero represent little to no genetic differentiation and values of one represent fixation of populations. The pairwise F_ST_ values calculated between the four major clusters ranged from 0.1125 to 0.24835, with the lowest pairwise F_ST_ being between the MCA and PSM clades and the highest being between the MCA and PM clades (Table 1).

**Table 1:** Weir and Cockerham unweighted pairwise F_ST_ values.

	AM	PM	PSM	MCA
**AM**	-			
**PM**	0.13283	-		
**PSM**	0.13972	0.21114	-	
**MCA**	0.15604	0.24835	0.1125	

Pairwise values calculated between clades of ingroup individuals.

**Fig 6:**
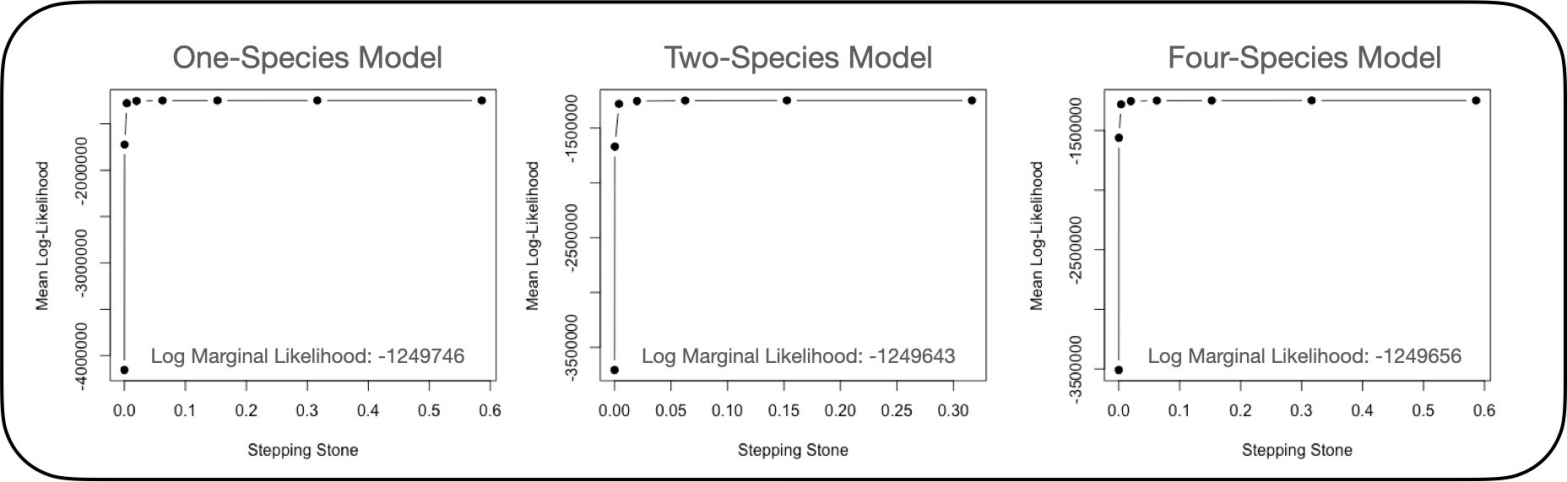
Species delimitation results for *Poeciliopsis*. Marginal likelihoods of the number of species in the ingroup sample calculated using the software BPP, and the R package bppr. Each point represents the mean log-likelihood calculated per stepping stone in BPP. The annotated likelihood in the lower right corner indicates the total marginal probability of the data. We used Bayes factor analysis to compare these marginal probabilities, concluding that the two-species model was more probable than the one-species model (BF = 1.00) and the four-species model (BF = 1.00).

Using BPP [72, 73], we recovered support for *P*. *gracilis* and *P*. *pleurospilus* as two separate species. The marginal likelihood of the data under the single-species model was -1,249,746 (Fig 6). The marginal probability of the data under the two-species model was -1,249,643, which per Bayes Factor comparison was significantly better than the one-species model (BF = 1.000). The marginal probability of the data under the four-species model was -1,249,656, which was significantly worse than the two-species model (BF = -1.000).

## Discussion

The family Poeciliidae represents one of the most ubiquitous groups of freshwater fishes in Middle America [45]. Species in the family have been heavily studied from both taxonomic and phylogenetic perspectives [21, 34, 36, 42, 46], using morphological and mitochondrial DNA data sets. Despite this flurry of activity, there remains much taxonomic uncertainty within the family. In many groups of organisms, inclusion of genomic sequence data has proven to be an informative approach to resolve relationships among taxa and identify evolutionary lineages among taxonomically difficult or unstable groups and recently diverged lineages [77–79]. Therefore, the overall goal of this study was to examine the taxonomic validity of *P*. *pleurospilus* and *P*. *gracilis* using analyses of genomic-scale sequence data in both phylogenetic and population genetic frameworks.

In the current literature, native populations of *P*. *pleurospilus* are hypothesized to inhabit Pacific Basin systems from Mexico to Honduras, as well as Atlantic Basin systems including the Río Grijalva (Mexico), upper Río Motagua (Guatemala), and Río Ulua basins (Honduras) [26, 45, 55]. Native populations of *P*. *gracilis* are hypothesized to be restricted to the Atlantic Basin in the states of Veracruz and Oaxaca, Mexico, as well as having been introduced in the Rios Pánuco (Atlantic) and Balsas (Pacific) within Mexico [45]. Based on the results of our phylogenetic, population genetic, and species delimitation analyses, we propose a distributional revision. *Poeciliopsis gracilis* consists of two lineages (AM and PM groups/clades) and their recognized distribution should be restricted to Atlantic basin streams from the Isthmus of Tehuantepec in Mexico and north in the states of Oaxaca and Veracruz and in Pacific basin streams from the Isthmus of Tehuantepec in Mexico, and north and west in the states of Oaxaca and Guerrero. Sister to these were a clade of *P*. *pleurospilus*, also with two lineages (PSM and MCA groups/clades). One lineage (PSM) was restricted to the Río Ostuta, a Pacific basin stream from Oaxaca, Mexico. The other lineage of *P*. *pleurospilus* (MCA) consists of individuals from both Pacific and Atlantic streams south and east of the Isthmus of Tehuantepec, from Chiapas, Mexico to Honduras, and include all specimens from all rivers in Guatemala and El Salvador.

As stated earlier, *P*. *gracilis* was described from the Río Orizaba on the Atlantic Slope of Veracruz, Mexico [51]. The Río Orizaba is a tributary to the Rio Blanco, which is the sampling location of nine individuals used in this study. These samples grouped in the AM cluster/clade in the results of all of our phylogenetic and population genetic analyses, indicating that this cluster/clade represent topotypic *P*. *gracilis*. The other samples that grouped in this cluster/clade were all north and west of the Isthmus of Tehuantepec, on both the Pacific and Atlantic slopes of Mexico. The samples from the AM clade were sister to the PM clade with 91% ultrafast bootstrap support and 99.6% SH-aLRT support. The population genetic analyses both obtained two distinct clusters north and west of the Isthmus of Tehuantepec.

*Poeciliopsis pleurospilus* was originally described from Lago Dueñas on the Pacific slope of Guatemala [52]. The historical location of this lake is approximately 12 km from the headwaters of the Río Achiguate, which was the sampling location of two samples used in this study. These samples grouped in the MCA cluster/clade in all phylogenetic and population genetic analyses, indicating that this cluster/clade represents individuals of topotypic *P*. *pleurospilus*. The other samples that were sister to this clade in the phylogenetic analysis were all south of the Isthmus of Tehuantepec, in southern Mexico, El Salvador, Guatemala, and Honduras. The samples from the PSM cluster/clade consistently were sister to the MCA clade in all of the phylogenetic and population genetic analyses, with the phylogenetic analysis having 92% ultrafast bootstrap support and 100% SH-aLRT support values for these two clades. The population genetic analyses also support the existence of two distinct clusters within this species.

Despite the fact that both *P*. *gracilis* and *P*. *pleurospilus* were described more than 150 years ago [51, 52], these two species have long remained taxonomically ambiguous and multiple reasons account for this. First, no comprehensive morphological or molecular study of either *P*. *gracilis* or *P*. *pleurospilus* has been published to date. Miller et al. 2005 [45] describes different pigmentation patterns to differentiate these species, but our observations suggested that these may not be reliable indicators. Additional research is needed to quantify pigmentation patterns between *P*. *gracilis* and *P*. *pleurospilus*. Although both species are widespread and abundant, the lack of studies attempting to refute or support their taxonomic validity has led to continued taxonomic uncertainty for these species. Next, throughout Middle America, non-native introductions of freshwater fishes have been rampant, further leading to taxonomic uncertainty. For example, several poeciliids (i.e., *Gambusia*, *Poecilia*, and *Xiphophorus* spp.) have been introduced for mosquito control and have successfully colonized over 40 countries [53, 79]. Additionally, poeciliids have been incidentally released during the stocking of tilapia (*Oreochromis* and *Tilapia spp*.), which have been introduced into multiple water bodies throughout Mexico and Central America [29, 80]. Native poeciliids often inhabit stock ponds with tilapia and then are inadvertently introduced into other drainage basins as tilapia are stocked and their presence often confounds the process of making robust taxonomic identifications in the field(K. Piller and C. McMahan, *pers*. *obs*.).

Finally, an additional regional aspect to consider is the active history of inter-basin hydrological exchanges from headwater stream capture or across the flood plains of lowlands in Middle America. The area near the Isthmus of Tehuantepec represents one of the lowest elevation points between the Atlantic and Pacific basins in the New World. Many of the headwater reaches of Atlantic and Pacific basin streams come into close proximity in the region, and this situation may have permitted inter-basin exchanges of populations during rainy seasons or flooding events, from modern to recent geological times. In addition, it is well known that some species of freshwater fishes can tolerate low salinities and are therefore able to disperse along the flood plains of lowland regions during ecologically appropriate time periods [15, 17] and this is particularly true for some species of poeciliids, which can often be found in low salinity habitats [81]. All of these factors together increase the likelihood that the neotropical freshwater ichthyofauna may have much wider distributions than originally proposed, especially for generalist species such as *Poeciliopsis*, and leaves room for distributional revisions for many other taxa as new data are gathered.

The results of this study are consistent with multiple other studies that have demonstrated the Isthmus of Tehuantepec to be an important biogeographical region where major changes occur in the distributional patterns of many groups [14, 82–86]. These distributional changes may stem in part from the fact that the Isthmus of Tehuantepec represents a geologically complex zone that has been subjected to various tectonic events, which in turn have changed the environmental conditions available to organisms, such as sea-level changes connecting or isolating various aquatic systems [86]. In addition to being a region that may have facilitated headwater river capture events during high-water periods or under changing erosional conditions, the Isthmus may also have acted as a geologic barrier that separated *P*. *gracilis* and *P*. *pleurospilus* populations, as has been demonstrated in a variety of other taxa [83, 87–89].

## Conclusions

*Poeciliopsis gracilis* and *P*. *pleurospilus* are morphologically and ecologically similar species of live-bearing freshwater fishes, which have had a widely debated taxonomic history and geographic distribution. This study presented comprehensive genomic evidence that was analyzed in both a phylogenetic and population genetic framework to shed light on the taxonomic status of both species. The results from phylogenetic, population genetic, and species delimitation analyses showed clear evidence that individuals of *P*. *gracilis* are distributed from the Isthmus and Tehuantepec and north in Atlantic basin systems in Mexico, whereas individuals of *P*. *pleurospilus* are distributed in both Atlantic and Pacific basin systems south and east of the Isthmus of Tehuantepec, from southern Mexico to Honduras.

## Supporting information

S1 Appendix Laboratory protocol. (DOCX)

S2 Appendix Ipyrad summary statistics. (TXT)

S3 Appendix Bioinformatic workflow. (DOCX)

S4 Appendix Treefile of phylogenetic inference. (TREEFILE)

S1 Fig A-score optimization–spline interpolation. (TIF)

S1 Table Sampling localities of ingroup individuals. (DOCX)

S2 Table Sampling localities of outgroup individuals. (DOCX)

S3 Table Barcodes and batch information. (DOCX)

S4 Table Parameters used for assembly of concatenated dataset. (DOCX)
