# Supplementary material for: Genomic data support the taxonomic validity of Middle American livebearers Poeciliopsis gracilis and Poeciliopsis pleurospilus (Cyprinodontiformes: Poeciliidae)
Source: PLoS One. 2022 Jan 31;17(1):e0262687. doi: 10.1371/journal.pone.0262687 (PMC8803166; doi:10.1371/journal.pone.0262687)
Supplement: S1 Appendix — (DOCX) [file pone.0262687.s001.docx]

**S1 Appendix. Laboratory protocol.**

**Dilution of PstI adapters – from 100uM to 1uM**

1. UV all equipment and buffers before starting.
2. Keep everything on ice throughout.
3. Make a spreadsheet of acquired oligos for book keeping purposes.
4. Use a semi-skirted plate; label appropriately- “1uM PstI adapters ‘date’”.
5. Pipette 120uL of Buffer AE (from Qiagen DNA extraction kit) into each well of the 96 well plate.
6. Place the plate containing the 1uM oligos into the thermocycler with the following conditions:
   1. 1 X 95°C 3:00 min
   2. 65 X 95°C 1:00 min (decreasing 1°C every cycle)
      1. Will end at 30°C
   3. Hold at 4°C
7. Next Qubit (BR kit) each of the 96 barcodes and record them in the spreadsheet made earlier – large range in values (50-1000 ng/uL).
8. Oligos of different length have different molarity, therefore different values must be used to calculate proper dilutions to 1uM. Follow this table to help calculate how much template to add in order to make a 250uL stock of 1uM oligos.

| Length | ng/uL for 1uM | Concentration (ng/uL) | Formula |
| --- | --- | --- | --- |
| 7 | 16.2 | W | (250*16.2)/W |
| 8 | 16.8 | X | (250*16.8)/X |
| 9 | 17.4 | Y | (250*17.4)/Y |
| 10 | 18.0 | Z | (250*18.0)/Z |

1. After a stock plate of 250uL of the 1uM is made, aliquot 9 uL of each barcode to a new 96 well plate. 9uM so that each plate will only be thawed twice before it is unusable (each plate uses 4uL of barcodes). Make as many aliquots as possible so that the stock plate does not have to be thawed as often.
2. Store all plates at -20°C for long-term use. Mark plates with a tick mark after it has been thawed.

**Dilution of MspI adapters – from 100uM to 10uM**

1. UV all equipment and buffers.
2. Keep everything on ice throughout.
3. Label a 1.5 mL tube “MspI 10uM”
4. Pipette 960 uL of Buffer AE (from Qiagen DNA extraction kit) to the 1.5mL tube.
5. Pipette 120uL of the TOP and BOTTOM MspI adapters to the tube (total of 1200 uL). Mix with pipette thoroughly; or briefly vortex.
6. Pipette the now 10uM common adapter evenly into multiple (3-4) strip tubes.
7. Place strip tubes in thermocycler and use the same protocol as the PstI oligos.
8. Aliquot out into multiple sets of strip tubes to avoid freeze thaw of oligos.

**Normalizing samples for Digestion**

1. All samples need to be roughly same concentration and volume prior to digestion.
2. In excel make a column for how much volume is need for each sample to have 300ng of DNA –

this will be different for each sample depending on the concentration.

1. Put that volume of each sample into a properly labeled 1.5mL tube.
2. All samples need to be at 17.2uL for the restriction enzyme digestion
   1. Add nuclease free water to samples with less than 17.2uL to adjust the volume
   2. Dry samples with greater than 17.2uL until the volume is approximately 18uL.
3. Place tubes, with the lids open, into the vacufuge and run with medium heat.
4. Check the vacufuge periodically to see if any samples are down to the 18uL mark. Once a sample is at the correct volume, take it out of the vacufuge and replace it with a sample that has yet to be dried.
5. Transfer 17.2 uL of each sample into a semi skirted 96 well plate (keep track of which well holds which sample).

**Restriction Enzyme Digestion**

1. After samples are roughly same concentration and volume, continue to the digestion step.
2. Keep everything on ice.
3. Make a master mix of the following reagents:

| Reagents | Per Reaction (uL) | Total |
| --- | --- | --- |
| 10X NEB Cutsmart Buffer | 2.0 | 212 uL |
| PstI HF (20,000 U/mL) | 0.4 | 42.4 uL |
| MspI (20,000 U/mL) | 0.4 | 42.4 uL |

1. Make enough master mix for 106 samples (ten more than the plate of 96 samples)
2. Add 2.8uL of the master mix to each of the 96 samples (total of 20uL with 17.2 uL of DNA).
3. Cover the plate with strip tube caps, vortex briefly, and spin down in plate spinner.
4. Place the plate in the thermocycler with the following conditions:
   1. Hold at 37°C for 16 hours
5. Ligation works best if done soon after the enzyme digestion.
6. Store digestion product plate in fridge if necessary – DO NOT FREEZE.

**Ligation of Barcodes**

1. Perform soon after digestion if possible.
2. Keep everything on ice.
3. Make a master mix of the following reagents:

| Reagent | Per Reaction (uL) | Total (120) |
| --- | --- | --- |
| T4 DNA Ligase | 1.28 | 153.6 |
| T4 DNA Ligase Buffer | 4 | 480 |
| Nuclease free H2O | 9.72 | 1,166.4 |

1. Add 15uL of the master mix to each of the 96 digested DNA samples.
2. Add 1 uL of 10uM MspI adapters to each of the 96 wells.
3. Add 4uL of the specific PstI adapters to the corresponding wells.
4. Cover with strip tube caps, briefly vortex, and spin down samples in plate spinner.
5. Put plate in thermocycler with the following conditions:
   1. 22°C for 3 hours
   2. Hold at 4°C

**Quality Control Check Point**

1. Run digestion/ligation products on a gel.
2. Use 1uL of template DNA and 2.5uL of dye.
3. Run at about 80-100 volts on a 1% agarose gel (200mL gel)
4. A good run should show a smear of DNA with very little high molecular weight bands- if a lot of high weight DNA is present, consider digesting and ligating those samples again.

**Pooling**

1. Pool samples into 24 pools.
2. Pipette 25uL of each sample (4 samples) into an appropriately labeled 1.5mL tube (total of 100uL in each pool).
3. Results in a total of 24 pools with 100uL each.

**QIAquick PCR Purification Clean Up**

*This is a modified version of the protocol that comes with the kit*

1. Add 1:250 of the pH indicator to the PB Buffer.
2. Place EB Buffer (elution buffer) in hot water bath ~40°C
3. Add 5x as much PB Buffer as DNA.
   1. *Example: with 100uL of DNA, add 500uL of PB Buffer to each sample.*
4. Add 10uL of 3M Sodium Acetate to each tube and vortex briefly.
5. Pipette each sample into labeled spin column tubes.
6. Centrifuge at 18000 rcf for 1 minute.
7. Pipette 750uL of PE Buffer to each sample.
8. Centrifuge at 18000 rcf for 1 minute.
9. Without adding anything, centrifuge again at 18000 rcf
   1. Discard flow-through and place spin columns in a labeled 1.5 mL tube.
10. Add 20uL of warm EB buffer directly to the filter. Do not let buffer touch the sides of the tube. Let EB buffer sit for 5 minutes on the filter (preferably in the hot water bath).
11. Centrifuge at 18000 rcf for 1 minute.
12. Elute again with 10uL of EB Buffer following the same directions as the previous step.
13. There should be roughly 30uL of sample in each of the 24 pools.

**PCR**

1. Qubit all 24 samples and use excel to calculate the volume is needed for about 200ng of DNA from each pool to be in the PCR step.
2. Subtract the amount of DNA from 35uL of water
   1. *Example: if 10 uL of DNA is needed, only add 25uL of water to the reaction.*
3. Create a master mix of the following reagents:

| Reagent | Per Reaction (uL) |
| --- | --- |
| 5X Phusion Buffer HF | 10 |
| 10mM DNTP’s | 1 |
| PstI primer (10mM) | 1 |
| MspI primer (10mM) | 1 |
| DMSO | 1.5 |
| Phusion Polymerase | 1 |

1. Add appropriate amount of water into each tube first (based on concentration).
2. Add 15.5 uL of the master mix to each sample.
3. Add the DNA for a total of 50.5uL reaction volume.
4. Place tubes in the thermocycler with the following conditions:
   1. 1 X 98°C – 30 sec
   2. 12 X 98°C – 30 sec, 62°C – 30 sec, 72°C – 30 sec
   3. 1 X 72°C – 10 min.
   4. Hold at 4°C.
5. Run a gel of all 24 samples to see that there is a smooth smear from the 100-1000 bp range.

**Post PCR Pooling and Cleanup**

1. Qubit the 24 samples again and normalize into 4 pools so that each pool has 2000 ng of DNA.
2. Perform the QiaQuick PCR purification cleanup on each of the 4 samples (amount of Buffer PB will vary based on volume of each pool).
3. Qubit the 4 pools and make a final pool with 1200ng of DNA from each pool (total 4800 ng).
4. Perform one last PCR purification clean up on the final library and elute with 40uL (20 + 20). Qubit one final time to know the concentration and amount of DNA.
