# Supplementary material for: Genomic data support the taxonomic validity of Middle American livebearers Poeciliopsis gracilis and Poeciliopsis pleurospilus (Cyprinodontiformes: Poeciliidae)
Source: PLoS One. 2022 Jan 31;17(1):e0262687. doi: 10.1371/journal.pone.0262687 (PMC8803166; doi:10.1371/journal.pone.0262687)
Supplement: S3 Appendix — (DOCX) [file pone.0262687.s003.docx]

**S3 Appendix. Bioinformatic Workflow.** Contains code for running FastQC quality control, ipyrad assembly, VCFtools filtering, IQ-TREE model finder and phylogenetic inference, DAPC and Composite Stacked Bar Plot, and calculation of F_ST_ values.

**## FastQC**

#Download FastQC and upload it to a cluster, then navigate to the folder

cd FastQC
chmod +x fastqc
./fastqc ../pathtodata.fastq.gz

#Makes FastQC executable and runs the program

**## ipyrad**

wget https://repo.continuum.io/miniconda/Miniconda3-latest-Linux-x86_64.sh
bash Miniconda3-latest-Linux-x86_64.sh
conda install ipyrad -c conda-forge -c bioconda
conda create -n ipyrad
conda activate ipyrad
conda install ipyrad -c conda-forge -c bioconda
conda config --add channels conda-forge
conda config --set channel_priority strict

#Installs and activates Miniconda and ipyrad

ipyrad -n plateonedata
nano params-plateonedata.txt

#Creates a parameters file for first plate
#Add path to data and barcodes

ipyrad -p params-plateonedata.txt -s1

#Starts step 1 of ipyrad pipeline – demultiplexes data and assigns barcodes

ipyrad -p params-fileyouwanttosplit.txt -b nameofnewfile samplename1 samplename2

#Use this to split any samples off a plate if necessary

ipyrad -m params-merged.txt params-file1.txt params-file2.txt

#Use this to merge any plates/samples if necessary

ipyrad -p params-merged.txt -s2

#Assigns each read a quality score

ipyrad -p params-alldata.txt -s3

#Clusters samples by individual

ipyrad -p params-alldata.txt -s4

#Examines heterozygosity and error rate

ipyrad -p params-alldata.txt -s5

#Builds the consensus

ipyrad -p params-alldata.txt -s67

#Re-examines clustering and writes file out to a useable format

**## VCFtools**

#Download VCFtools from https://sourceforge.net/projects/vcftools/ and upload it to the cluster
#Navigate to vcftools file on cluster

chmod +x vcftools_0.1.13.tar.gz
tar -xvf vcftools_0.1.13.tar.gz
cd vcftools_0.1.13
make
cd vcftools_0.1.13
cd bin

#Makes VCFtools executable

./vcftools --vcf pathtodata.vcf --missing-indv
view out.imiss

#Lists how much data each sample is missing

nano samplestoremove.indv

#Make a list of individuals to be removed

./vcftools --vcf pathtofile.vcf --remove samplestoremove.indv --max-missing 0.6 --recode --out nameofnewfile

#Removes individuals from previous step and filters out loci missing more than 60%

**## IQ-TREE**

iqtree -s fastafile.fa
iqtree -s example.phy -m MFP

#Runs iqtree Model Finder to find the best fit model for the data

iqtree -s fastafile.fasta -m GTR+F+R2 -alrt 1000 -bb 10000

#Runs a maximum likelihood phylogenetic inference with 1000 SH-aLRT replications and 10,000 ultrafast bootstrap iterations

**## DAPC analysis and Composite Stacked Bar Plot**

library(vcfR)

library(poppr)

library(ape)

library(RColorBrewer)

#Loads required packages

rubi.vcf <- read.vcfR("ingroup.recode.vcf")

pop.data <- read.table("populations.txt", sep = "\t", header=TRUE)

all(colnames(rubi.vcf@gt)[-1] == pop.data$AccessID)

#Loads VCF data and population information

gl.rubi <- vcfR2genlight(rubi.vcf)

ploidy(gl.rubi) <- 2

pop(gl.rubi) <- pop.data$Population

#Converts the data to a genlight object

dapc1 <- dapc(gl.rubi)

Choose the number PCs to retain (>=1):

8

Choose the number discriminant functions to retain (>=1):

3

#Inital DAPC based on x-value PC retention

optim.a.score(dapc1)

#Provides an a-score to fine-tune PC retention values

dapc2 <- dapc(gl.rubi)

Choose the number PCs to retain (>=1):

6

Choose the number discriminant functions to retain (>=1):

3

#Re-runs the DAPC to update the number of PCs to retain

myCol <- c("orange", "blue", "red", "darkgreen")

scatter(dapc2, scree.da = TRUE, posi.da = "topleft", scree.pca = TRUE, posi.pca = "topright", bg="white", pch=20, col=myCol, clab=0, cex=1.75)

#Constructs scatterplot of DAPC with labels

dapc.results2 <- as.data.frame(dapc2$posterior)

dapc.results2$pop <- pop(gl.rubi)

dapc.results2$indNames <- rownames(dapc.results2)

dapc.results2 <- pivot_longer(dapc.results2, -c(pop, indNames))

colnames(dapc.results2) <- c("Original_Pop","Sample","Assigned_Pop","Posterior_membership_probability")

p <- ggplot(dapc.results2, aes(x=Sample, y=Posterior_membership_probability, fill=Assigned_Pop))

p <- p + geom_bar(stat='identity')

p <- p + scale_fill_manual(values = cols)

p <- p + facet_grid(~Original_Pop, scales = "free")

p <- p + theme(axis.text.x = element_text(angle = 90, hjust = 1, size = 8))

#Constructs a composite stacked bar plot

**## Calculations of F_ST_ values**

./vcftools --vcf ingroup.recode.vcf --weir-fst-pop AM.txt --weir-fst-pop PM.txt --out AM-PM_fst

#Compares Fst values between the AM and PM clusters

./vcftools --vcf ingroup.recode.vcf --weir-fst-pop AM.txt --weir-fst-pop PSM.txt --out AM-PSM_fst

#Compares Fst values between the AM and PSM clusters

./vcftools --vcf ingroup.recode.vcf --weir-fst-pop AM.txt --weir-fst-pop MCA.txt --out AM-MCA_fst

#Compares Fst values between the AM and MCA clusters

./vcftools --vcf ingroup.recode.vcf --weir-fst-pop PM.txt --weir-fst-pop PSM.txt --out PM-PSM_fst

#Compares Fst values between the PM and PSM clusters

./vcftools --vcf ingroup.recode.vcf --weir-fst-pop PM.txt --weir-fst-pop MCA.txt --out PM-MCA_fst

#Compares Fst values between the PM and MCA clusters

./vcftools --vcf ingroup.recode.vcf --weir-fst-pop PSM.txt --weir-fst-pop MCA.txt --out PSM-MCA_fst

#Compares Fst values between the PSM and MCA clusters
