# Supplementary figures and images for: Genomic data support the taxonomic validity of Middle American livebearers Poeciliopsis gracilis and Poeciliopsis pleurospilus (Cyprinodontiformes: Poeciliidae)
Source: PLoS One. 2022 Jan 31;17(1):e0262687. doi: 10.1371/journal.pone.0262687 (PMC8803166; doi:10.1371/journal.pone.0262687)

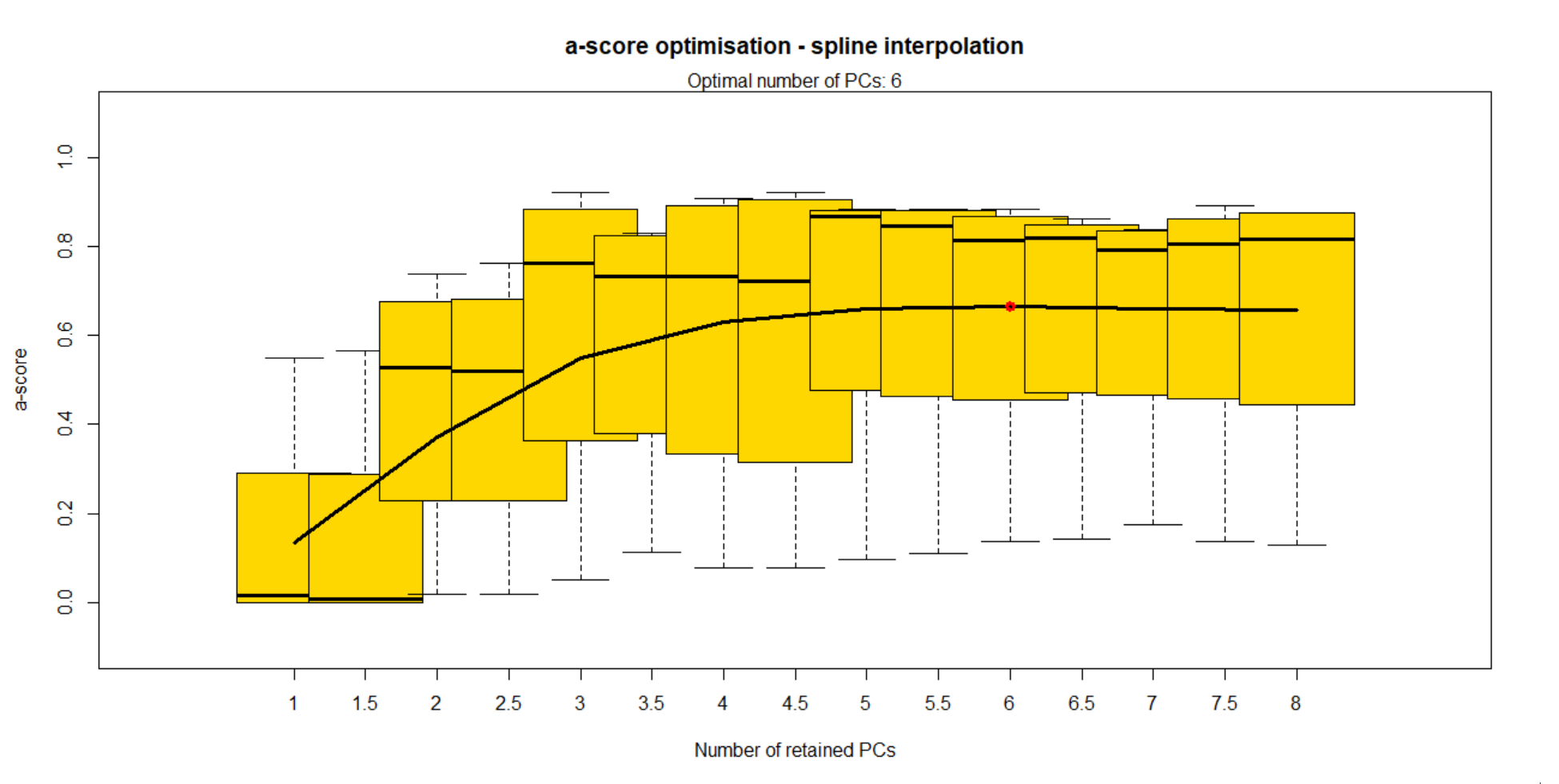

Supplement: S1 Fig — (TIF) [file pone.0262687.s005.tif]
