# Supplementary material for: Genomic data support the taxonomic validity of Middle American livebearers Poeciliopsis gracilis and Poeciliopsis pleurospilus (Cyprinodontiformes: Poeciliidae)
Source: PLoS One. 2022 Jan 31;17(1):e0262687. doi: 10.1371/journal.pone.0262687 (PMC8803166; doi:10.1371/journal.pone.0262687)
Supplement: S1 Table — (DOCX) [file pone.0262687.s006.docx]

**Table S1. Sampling localities of ingroup individuals.**

| **Specimen** | **Basin** | **Latitude** | **Longitude** | **Voucher** | **Locality** |
| --- | --- | --- | --- | --- | --- |
| **El Salvador** |  |  |  |  |  |
| ES_R | Pacific | 14.25 | -89.48 | LSUMZ-TC 2485 | Lago Guija, Depart. Santa Ana |
| ES_V | Pacific | 14.32 | -89.46 | LSUMZ-TC 2496 | Laguna Metapan, Depart. Santa Ana |
| **Guatemala** |  |  |  |  |  |
| Guat_B | Pacific | 14.27 | -90.90 | FMNH 130784.1 | Río Achiguate, Esquintla |
| Guat_C | Pacific | 13.88 | -90.00 | FMNH 130799.1 | Río Negro, Jutiapa |
| Guat_E | Pacific | 13.88 | -90.00 | FMNH 130799.2 | Río Negro, Jutiapa |
| Guat_F | Pacific | 14.27 | -90.90 | FMNH 130784.2 | Río Achiguate, Esquintla |
| **Honduras** |  |  |  |  |  |
| Hond_M | Pacific | 13.48 | -87.10 | LSUMZ-TC 4436 | Orocuina, Depart. de Choluteca |
| Hond_Q | Atlantic | 14.74 | -87.97 | LSUMZ-TC 3069 | Río Tepemechin, Depart. Comayagua |
| Hond_X | Atlantic | 14.91 | -89.01 | LSUMZ-TC 3108 | Río Amarillo, Depart. de Copan |
| Hond_Y | Pacific | 13.83 | -87.69 | LSUMZ-TC 3195 | Nacaome, Depart. de Valle |
|  |  |  |  |  |  |
| **Mexico** |  |  |  |  |  |
| Mex_01 | Atlantic | 16.20 | -92.19 | MZCP 116 | Río Ojo de Agua, Chiapas |
| Mex_11 | Pacific | 15.59 | -93.06 | MZCP 1310 | Río Margaritas, Chiapas |
| Mex_18 | Atlantic | 15.77 | -91.98 | MZCP 2421 | Río Paso Hondo, Chiapas |
| Mex_ 21 | Pacific | 16.33 | -95.24 | SLU 116 | Río Tehuantepec, Oaxaca |
| Mex_23 – 33, 35, 37 | Pacific | 16.50 | -94.44 | - | Río Ostuta, Oaxaca |
| Mex_39, 41 – 44 | Pacific | 16.34 | -95.24 | - | Río Tehuantepec, Oaxaca |
| Mex_ 46 – 47 | Pacific | 16.34 | -95.24 | - | Río Tehuantepec, Oaxaca |
| Mex_48 – 53 | Pacific | 16.41 | -95.60 | - | Río Tequesistlan, Oaxaca |
| Mex_55 – 57 | Pacific | 16.41 | -95.60 | - | Río Tequesistlan, Oaxaca |
| Mex_58 – 66 | Pacific | 16.56 | -96.03 | - | Río de la Virgen, Oaxaca |
| Mex_68 – 71, 73, 77 | Pacific | 16.67 | -96.27 | - | Río Totolapan, Oaxaca |
| Mex_80 – 84, 86, 87 | Pacific | 16.79 | -96.67 | - | Río Octlan, Oaxaca |
| Mex_89, 92 – 101 | Pacific | 16.60 | -96.74 | - | Río Coapa, Oaxaca |
| Mex_103– 105 | Pacific | 16.60 | -96.74 | - | Río Coapa, Oaxaca |
| Mex_107 – 112 | Pacific | 16.60 | -96.74 | - | Río Coapa, Oaxaca |
| Mex_G02 | Pacific | 17.27 | -99.55 | UNICACH 319 | Unnamed Arroyo, Guerrero |
| Mex_G03 | Pacific | 17.04 | -97.91 | UNICACH 450 | Puente Tierra Azul, Oaxaca |
| Mex_G05 | Pacific | 16.35 | -97.09 | UNICACH 481 | Río Las Flores, Oaxaca |
| Mex_G08 | Atlantic | 19.40 | -96.65 | UNICACH 699 | Río del Plan, Veracruz |
| Mex_G1 | Pacific | 15.83 | -96.33 | - | Río Huatulco, Oaxaca |
| Mex_G6 | Atlantic | 16.10 | -97.07 | UNICACH 509 | Río Flor de Café, Oaxaca |
| Mex_G9, G11, G20 | Atlantic | 17.28 | -95.07 | - | Unnamed Arroyo, Oaxaca |
| Mex_G13 | Atlantic | 18.17 | -96.10 | - | Río Papaloapan, Oaxaca |
| Mex_G14, G15, G17 | Atlantic | 17.39 | -95.06 | - | Río Jaltepec, Veracruz |
| Mex_G19 | Pacific | 17.94 | -99.59 | - | Río Mezcala, Guerrero |
| Mex_G22 | Atlantic | 18.58 | -96.67 | - | Río Barranca, Oaxaca |
| Mex_G23 | Atlantic | 18.52 | -96.43 | - | Río Amapa, Oaxaca |
| Mex_G26 | Atlantic | 19.47 | -96.47 | - | Río Actopan, Veracruz |
| Mex_G36 – 38 | Atlantic | 17.20 | -95.05 | - | Río Tolosita, Oaxaca |
| Mex_G40 – 43, G45 | Atlantic | 17.20 | -95.05 | - | Río Tolosita, Oaxaca |
| Mex_G47 – G55 | Atlantic | 18.74 | -96.45 | - | Río Blanco, Veracruz |
| Mex_G57 – G65 | Atlantic | 17.20 | -95.05 | - | Río Tolosita, Oaxaca |
| Mex_G68 – G72 | Atlantic | 16.77 | -95.02 | - | Río Ajal, Oaxaca |
| Mex_G74, G76 | Atlantic | 16.77 | -95.02 | - | Río Ajal, Oaxaca |
