# Supplementary material for: Genomic data support the taxonomic validity of Middle American livebearers Poeciliopsis gracilis and Poeciliopsis pleurospilus (Cyprinodontiformes: Poeciliidae)
Source: PLoS One. 2022 Jan 31;17(1):e0262687. doi: 10.1371/journal.pone.0262687 (PMC8803166; doi:10.1371/journal.pone.0262687)
Supplement: S2 Table — (DOCX) [file pone.0262687.s007.docx]

| **Species** | **Specimen ID** | **Latitude** | **Longitude** | **Locality** |
| --- | --- | --- | --- | --- |
| *Brachyrhaphis rhabdophora* |  |  |  |  |
|  | brhab |  |  | N/A |
| *Poeciliopsis fasciata* |  |  |  |  |
|  | Mex_F1 – F5 | 16.77 | -95.02 | Río Ajal, Mexico |
| *Poeciliopsis infans* |  |  |  |  |
|  | pinfans_1 – 4 |  |  | Man. Mintzita, Mexico |
| *Poeciliopsis turrubarensis* |  |  |  |  |
|  | Mex_T10 | 16.34 | -95.24 | Río Tehuantepec, Mexico |
|  | Mex_T15 | 16.41 | -95.60 | Río Tequesistlan, Mexico |
|  | Mex_T20 | 16.56 | -96.03 | Río de la Virgen, Mexico |
|  | Mex_T25 | 16.67 | -96.27 | Río Totolapan, Mexico |

**S2 Table. Sampling localities of outgroup individuals.**
