# Supplementary material for: Genomic data support the taxonomic validity of Middle American livebearers Poeciliopsis gracilis and Poeciliopsis pleurospilus (Cyprinodontiformes: Poeciliidae)
Source: PLoS One. 2022 Jan 31;17(1):e0262687. doi: 10.1371/journal.pone.0262687 (PMC8803166; doi:10.1371/journal.pone.0262687)
Supplement: S3 Table — (DOCX) [file pone.0262687.s008.docx]

**S3 Table. Barcodes and batch information.** Unique PstI oligos used for ligation of ddRADseq samples and batch numbers for each of the specimens included in this study.

| **Location** | **Barcode** | **Batch 1** | **Batch 2** | **Batch 3** |
| --- | --- | --- | --- | --- |
| 1A | AACAATG | Mex_105 | Mex_G9 |  |
| 1B | CCACCGT | Mex_111 |  |  |
| 1C | TTGTTCA | Mex_110 | Mex_G14 | - |
| 1D | CGTGGAA | Mex_83 | Mex_G15 | - |
| 1E | GAACAAT | Mex_81 | - | - |
| 1F | ACCATCG |  | Mex_G23 | - |
| 1G | TTGGCTA | Mex_35 |  | - |
| 1H | ACTTGGT | Mex_66 | - | - |
| 2A | GGAACTG | Mex_93 | Mex_G11 | - |
| 2B | CACCAGT | Mex_108 | Mex_G13 | - |
| 2C | TGTTGCA | Mex_100 |  | - |
| 2D | CTGGTAG | Mex_99 | Mex_G20 | - |
| 2E | GAACCTA | Mex_41 | Mex_G22 | - |
| 2F | ACCAAGT | Mex_47 | Mex_G38 | - |
| 2G | ATTGTCG | Mex_52 | Mex_G49 | - |
| 2H | CGGTGAT | Mex_57 | Mex_G51 | - |
| 3A | TAACACG |  |  | - |
| 3B | GCCACTA |  |  | - |
| 3C | CTTGTGA | Mex_33 | Mex_G70 | - |
| 3D | TCGTGAG | Mex_96 | Mex_G74 | - |
| 3E | GGAATCT |  | Mex_G76 | - |
| 3F | AACCGAT |  | Mex_G05 | - |
| 3G | TGTGCGA | Mex_62 | Mex_G17 | - |
| 3H | CTGTATG | Mex_70 | - |  |
| 4A | GAACATCA |  |  | - |
| 4B | ACCATACG |  | - | - |
| 4C | TTGTCGCA | Mex_37 | Mex_G36 | - |
| 4D | AGTGGCCG | Mex_73 | Mex_G37 | - |
| 4E | GAACTACG | Mex_109 |  | - |
| 4F | CCTAAGCA | Mex_24 | Mex_G42 | - |
| 4G | TTCTGCCA | Mex_63 | Mex_G43 | - |
| 4H | GAGGCTCG |  |  | pinfans_1 |
| 5A | AGAACATA | Mex_98 | Mex_G45 | - |
| 5B | CCTCACAG | Mex_101 | Mex_G47 | - |
| 5C | TTCTTGGA |  | Mex_G48 | - |
| 5D | GGAGGTCG | Mex_112 | Mex_G57 | - |
| 5E | AAGAACTA | Mex_64 | Mex_G58 | - |
| 5F | CCTCCACG | Mex_53 | Mex_G59 | - |
| 5G | TTCTGTAG | Mex_51 | Mex_G61 | - |
| 5H | GCGGTGGA | Hond_Y | Mex_G62 | pinfans_2 |
| 6A | AGAACTAG | Mex_97 | Mex_G63 | - |
| 6B | CACCTAGA | Mex_23 | Mex_G64 | - |
| 6C | TTGTACCG | Mex_92 | Mex_G65 | - |
| 6D | GGTCGGTA | Mex_27 | Mex_G68 | - |
| 6E | AACGAATA | Mex _86 | Mex_G71 | - |
| 6F | CCAACTCG | Mex _43 | Mex_G72 | - |
| 6G | TTGGTCAA | Mex _49 |  | - |
| 6H | CGTTGAGG | Mex _60 | Mex_G03 | pinfans_3 |
| 7A | GAACAGTCA |  | Mex_G40 | - |
| 7B | ACCACTGCA |  | Mex_G41 | - |
| 7C | TTGTTGACA | Mex _29 | Mex_G53 | - |
| 7D | GGTGGACCA | Mex _25 | Mex_G60 | - |
| 7E | AACATCGCA | Mex_77 | Mex_G69 | - |
| 7F | CCACGCTCA | Mex _46 |  | - |
| 7G | TTGTATCCA | Mex _59 | Guat_B | - |
| 7H | GGTGCGACA | Mex _61 | Guat_C | pinfans_4 |
| 8A | AACAAGTCA | Mex _89 | Mex_G1 | - |
| 8B | CCACCTGCA | Mex_31 | Mex_G6 | - |
| 8C | TTGGTACCA |  | Mex_G26 | - |
| 8D | GGTTGCACA | Mex _26 |  | - |
| 8E | AACAATAGA |  |  | - |
| 8F | CCACTAGAA |  | Mex_G50 | - |
| 8G | TTGTCCTCA | Mex _69 |  | - |
| 8H | GGTAGGCAA | Mex _58 | Mex_G52 | brhab |
| 9A | AACGAATGA | Mex _80 |  | - |
| 9B | CCACTGACA | Mex _107 | Mex_G08 | - |
| 9C | TTGTCTGAA | Mex _50 | Mex_G54 | - |
| 9D | CGTGGCCGA | Mex _44 |  | - |
| 9E | GAACCTCCA |  |  | - |
| 9F | ACCAACTGA | Mex _48 |  | - |
| 9G | TTGTGAGCA |  | Mex_G55 | - |
| 9H | CGTGTGAAT | ES_R |  | - |
| 10A | GAACACTACA | Mex _103 | Mex_G02 | - |
| 10B | ACCACTAGCA | Mex _28 |  | - |
| 10C | TTGTTAGCCA | Mex _82 | Mex_F1 | - |
| 10D | GGTAGGCACA | Mex _84 | Mex_F2 | - |
| 10E | AACGAATCCA |  | Mex_F3 | - |
| 10F | CCACCTAGCA | Mex_55 | Mex_F4 | - |
| 10G | TTGTTCCACA | Hond_X | Mex_F5 | - |
| 10H | GGTGAGGCCA |  |  | - |
| 11A | AACAGATGCA | Mex _87 | Mex_T10 | - |
| 11B | CCACTCCGCA | Mex _32 | Mex_T15 | - |
| 11C | TTGTCTAACA | Mex _42 | Mex_T20 | - |
| 11D | AGTGGAGCCA | Mex _94 | Mex_T25 | - |
| 11E | GAACCGTCCA | ES_V | Guat_E | - |
| 11F | CCTAACAGCA | Mex _30 | Hond_M | - |
| 11G | TTCTGTCACA | Mex _68 | Hond_Q | - |
| 11H | GAGGTGGACA | Mex _65 | Mex_01 | - |
| 12A | AGAACATCCA | Mex _104 | Guat_F | - |
| 12B | CCTCATCGCA |  |  | - |
| 12C | TTCTTCAGCA | Mex_71 |  | - |
| 12D | GTGGCGGCCA | Mex _95 | Mex_18 | - |
| 12E | AGAAGCTACA | Mex _39 |  | - |
| 12F | CACCATAGCA |  |  | - |
| 12G | TCTTGACACA | Mex _56 | - | - |
| 12H | GCGGTGGCAA | Mex _11 | Mex_21 | - |
