# Supplementary material for: Genomic data support the taxonomic validity of Middle American livebearers Poeciliopsis gracilis and Poeciliopsis pleurospilus (Cyprinodontiformes: Poeciliidae)
Source: PLoS One. 2022 Jan 31;17(1):e0262687. doi: 10.1371/journal.pone.0262687 (PMC8803166; doi:10.1371/journal.pone.0262687)
Supplement: S4 Table — (DOCX) [file pone.0262687.s009.docx]

| **Parameters** | **Parameters Used** |
| --- | --- |
| [assembly_method]: Assembly method (denovo, reference) | denovo |
| [datatype]: Datatype (see docs): rad, gbs, ddrad, etc. | rad |
| [restriction_overhang]: Restriction overhang (cut1,) or (cut1, cut2) | TGCAG, |
| [max_low_qual_bases]: Max low quality base calls (Q<20) in a read | 5 |
| [phred_Qscore_offset]: phred Q score offset (33 is default and very standard) | 33 |
| [mindepth_statistical]: Min depth for statistical base calling | 6 |
| [mindepth_majrule]: Min depth for majority-rule base calling | 6 |
| [maxdepth]: Max cluster depth within samples | 10,000 |
| [clust_threshold]: Clustering threshold for de novo assembly | 0.85 |
| [max_barcode_mismatch]: Max number of allowable mismatches in barcodes | 0 |
| [filter_adapters]: Filter for adapters/primers (1 or 2=stricter) | 0 |
| [filter_min_trim_len]: Min length of reads after adapter trim | 35 |
| [max_alleles_consens]: Max alleles per site in consensus sequences | 2 |
| [max_Ns_consens]: Max N's (uncalled bases) in consensus | 0.05 |
| [max_Hs_consens]: Max Hs (heterozygotes) in consensus | 0.05 |
| [min_samples_locus]: Min # samples per locus for output | 4 |
| [max_SNPs_locus]: Max # SNPs per locus | 0.2 |
| [max_Indels_locus]: Max # of indels per locus | 8 |
| [max_shared_Hs_locus]: Max # heterozygous sites per locus | 0.5 |
| [trim_reads]: Trim raw read edges (R1>, <R1, R2>, <R2) (see docs) | 0, 0, 0, 0 |
| [trim_loci]: Trim locus edges (see docs) (R1>, <R1, R2>, <R2) | 0, 0, 0, 0 |

**Table S4. Parameters used for assembly of concatenated dataset.**
